# Supplementary material for: Structural basis of the mycobacterial stress-response RNA polymerase auto-inhibition via oligomerization
Source: Nat Commun. 2023 Jan 30;14:484. doi: 10.1038/s41467-023-36113-y (PMC9886945; doi:10.1038/s41467-023-36113-y)
Supplement: Supplementary file 1 — Supplementary Information [file 41467_2023_36113_MOESM1_ESM.pdf]

Supplementary information

Structural basis of the mycobacterial stress-response RNA polymerase auto-inhibition via oligomerization

Morichaud et al.

Supplementary Table 1 Cryo-EM data collection, refinement and validation statistics

	Octamer D_4-map	Protomer	Dimer	Dimer class 1	Dimer class 2	Core	Octamer D_4-map	Protomer
**Data collection**								
Pixel size (Å)	1.1							
Voltage (kV)	300							
Electron dose (e^{-1} Å $^{-2}$)	49.6							
Defocus range (µm)	0.5 - 5							
**Reconstruction software**	CryoSPARC						RELION	
Total particles	254 380	254 380	254 380	254 380	254 380	721 752	157 386	753 528
Particles used in reconstruction	115 457	115 112	115 112	66 519	48 593	55 008	94 191	267 457
Symmetry imposed	D_4	-	-	-	-	-	D_4	-
Map resolution (Å) FSC threshold 0.143	4.4	3.84	4.36	4.38	6.75	4.08	6.3	3.86
**Refinement**								
Resolution FSC threshold 0.5	9.00	4.00	5.42	-	-	4.39	-	3.9
Map CC (whole map)	0.52	0.8	0.69	-	-	0.72	-	0.84
Map CC (peaks)	0.46	0.77	0.65	-	-	0.69	-	0.75
**RMSD**								
Bond length (Å)	0.003	0.002	0.003	-	-	0.003	-	0.007
Bond angle (°)	0.555	0.483	0.633	-	-	0.525	-	1.010
**Ramachandran plot**								
Preferred regions (%)	96.45	96.68	96.42	-	-	95.90	-	94.34
Allowed regions (%)	3.55	3.32	3.58	-	-	4.03	-	5.66
Outliers (%)	0.00	0.00	0.00	-	-	0.07	-	0.00
**Validation**								
MolProbity score	1.56	1.50	1.56	-	-	1.56	-	2.50
All-atom clashscore	5.90	5.44	5.91	-	-	5.19	-	10.37
Rotamer outliers (%)	0.71	0.39	0.70	-	-	0.37	-	5.77
EM accession	EMD-13817	EMD-13579	EMD-13829	EMD-14378	EMD-14974	EMD-14560	EMD-14697	EMD-14696
PDB accession	7Q4U	7PP4	7Q59	-	-	7Z8Q	-	7ZF2

FSC, Fourier shell correlation; RMSD, root mean square deviation

Supplementary Table 2 Sequences of primers used for DNA amplification and cloning

Primer name	Primer sequence 5'→ 3'	Product
SigAP_forw	[6FAM]CACTGAAACTTGCCCGCTC	106 bp *sigA*P promoter fragment labeled at the 5' end of non-template DNA strand
SigAP_rev	GCGTTGATATCAGCGTGAAT	
SigAP_TGTG +2G-Cy3 forw	CACTGAAACTTGCCCGCTCGGGCTGTACTCGTGCGCAGT TGTGTTACAATGGTCAGC[G-Cyanine3]GCGGC	106 bp *sigA*P promoter fragment internally labeled at +2G of non-template DNA strand
SigAP_TGTG rev	GCGTTGATATCAGCGTGAATACTCGCGCGCTATCGGTCGG GCGGCCGCCGCTGACCATTGTAACACAACTGCG	
CarD cloning *Nde*I forw	ggcagcatATGATTTTCAAGGTCGGAGACACCG	486 nt CarD gene (Rv3583c)
CarD cloning *Hind III* rev	gtcgcaagctttAAGACGCGGCGGCTAAAACCTC	

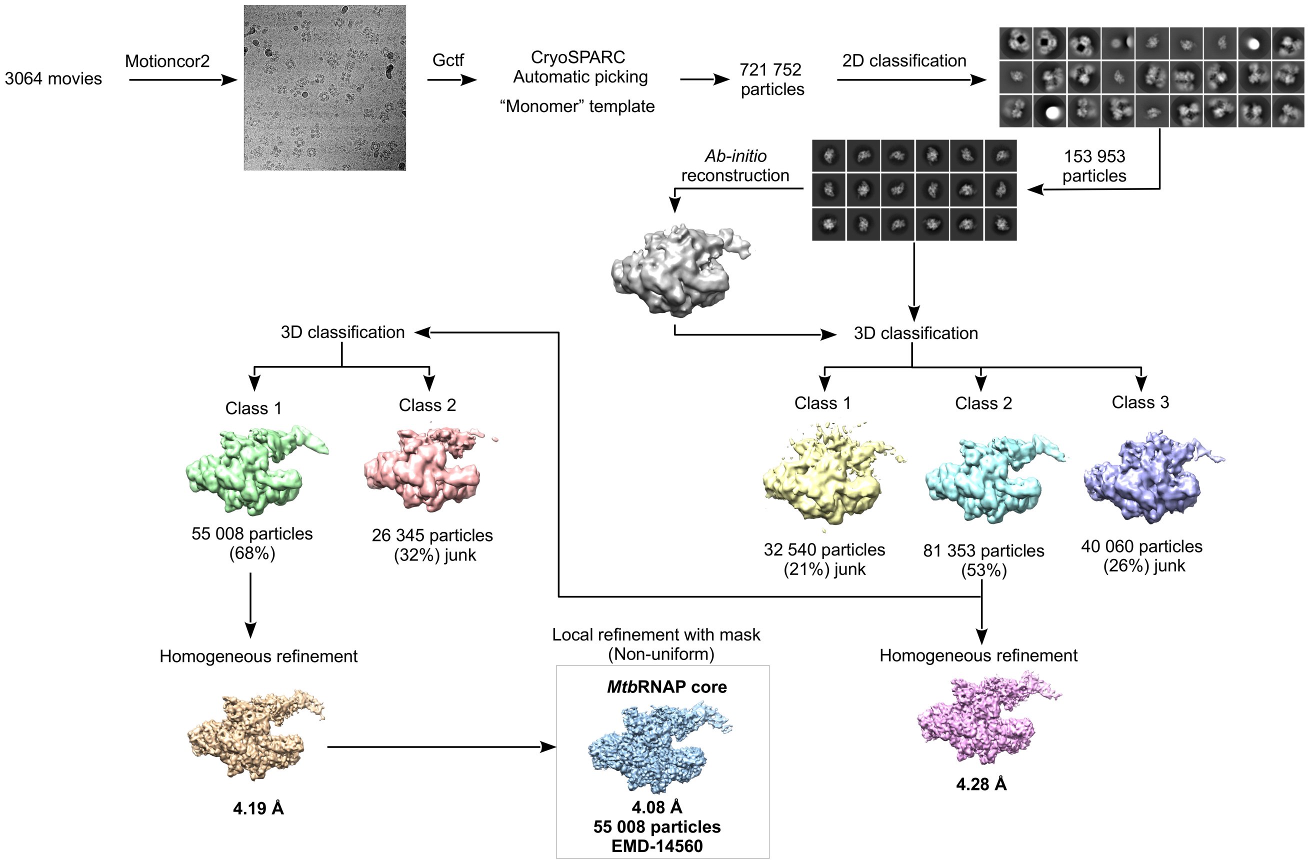

Supplementary Figure 1. The cryo-EM data processing pipeline for the *M. tuberculosis* RNAP core

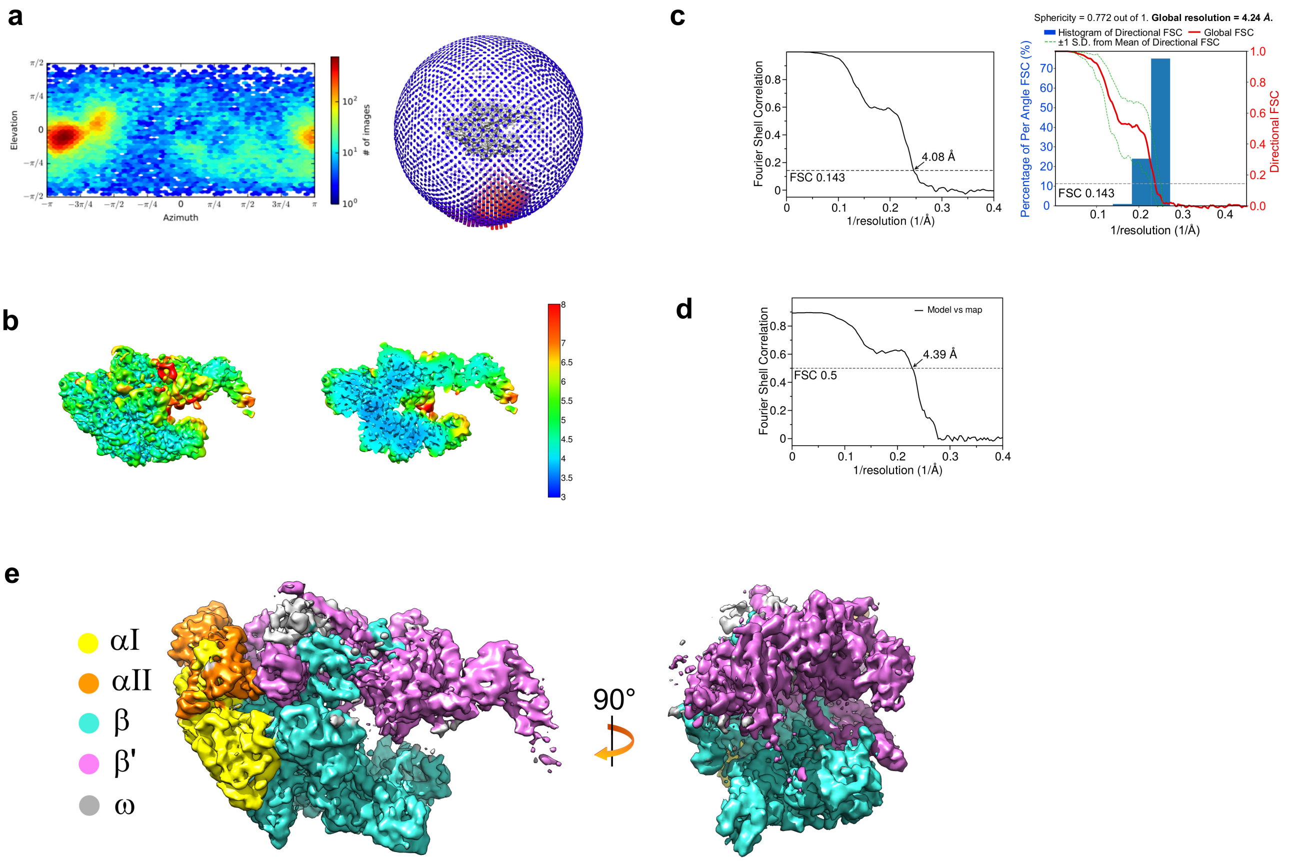

Supplementary Figure 2. The cryo-EM structure of the *M. tuberculosis* RNAP core. a Heat map and spherical presentation of the angular distributions for particles projections calculated in cryoSPARC. **b** Cryo-EM density map and sliced map (on the right) colored according to the local resolution. **c** Gold-standard Fourier Shell Correlation (FSC) calculated for the cryo-EM map in cryoSPARC. The dotted line shows the 0.143 FSC cutoff. Histogram (blue) and directional FSC Plot (red line) calculated for the cryo-EM map using on-line application at https://3dfsc.salk.edu/[1]. **d** Gold-standard FSC calculated for the model vs. full-map using the MTRIAGE module of Phenix (right). The dotted line shows the 0.5 FSC cutoff. **e** Views on the cryo-EM density map of the Mtb RNAP core. The RNAP subunits color-coded as indicated on the left.

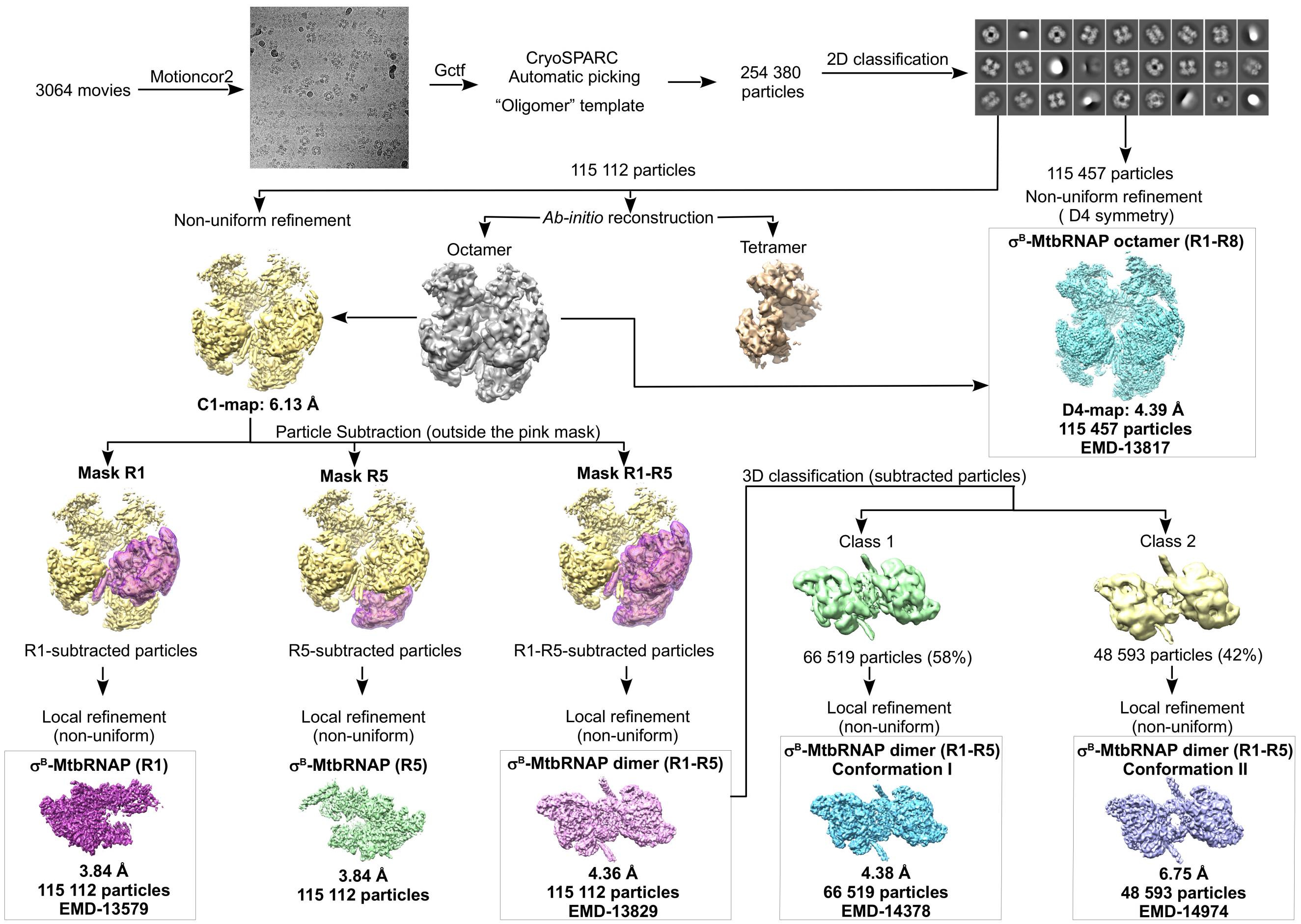

Supplementary Figure 3. The cryo-EM data processing pipeline for *M. tuberculosis* Eσ^B

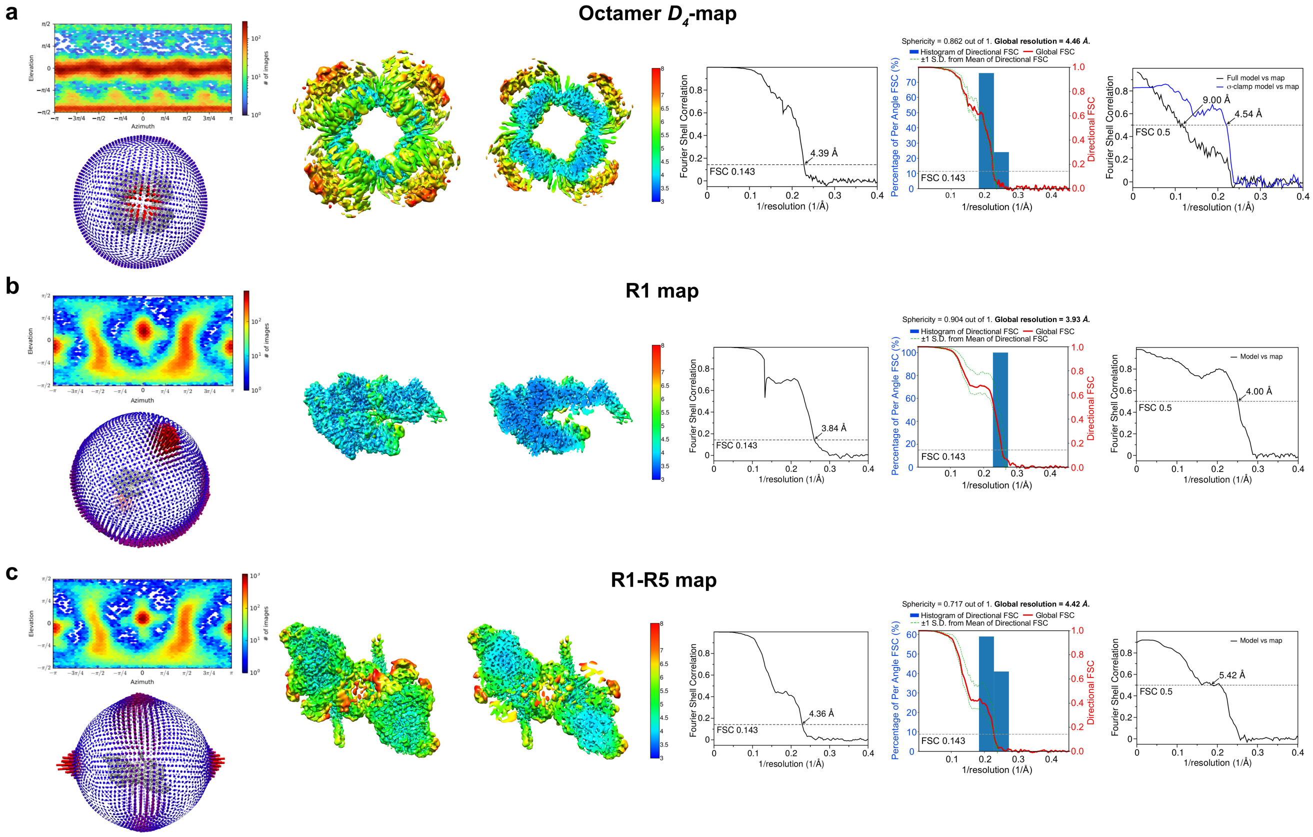

Supplementary Figure 4. ***M. tuberculosis*** **$E\sigma^{B}$ octamer, monomer and dimer. a** $E\sigma^{B}$ octamer. **b** $E\sigma^{B}$ R1 protomer. **c** $E\sigma^{B}$ R1-R5 dimer. From left to right: angular distributions for particles projections calculated in cryoSPARC and presented as a heat map and as a sphere: Cryo-EM density maps and sliced maps (on the right) colored according to the local resolution. Gold-standard FSC calculated for the map in cryoSPARC. The dotted line shows the 0.143 FSC cutoff. Histogram (blue) and directional FSC Plot (red line) calculated for the cryo-EM map using on-line application at https://3dfsc.salk.edu/[1]. Gold-standard FSC calculated for the model vs. full-map using the MTRIAGE module of Phenix (right). The dotted line shows the 0.5 FSC cutoff.

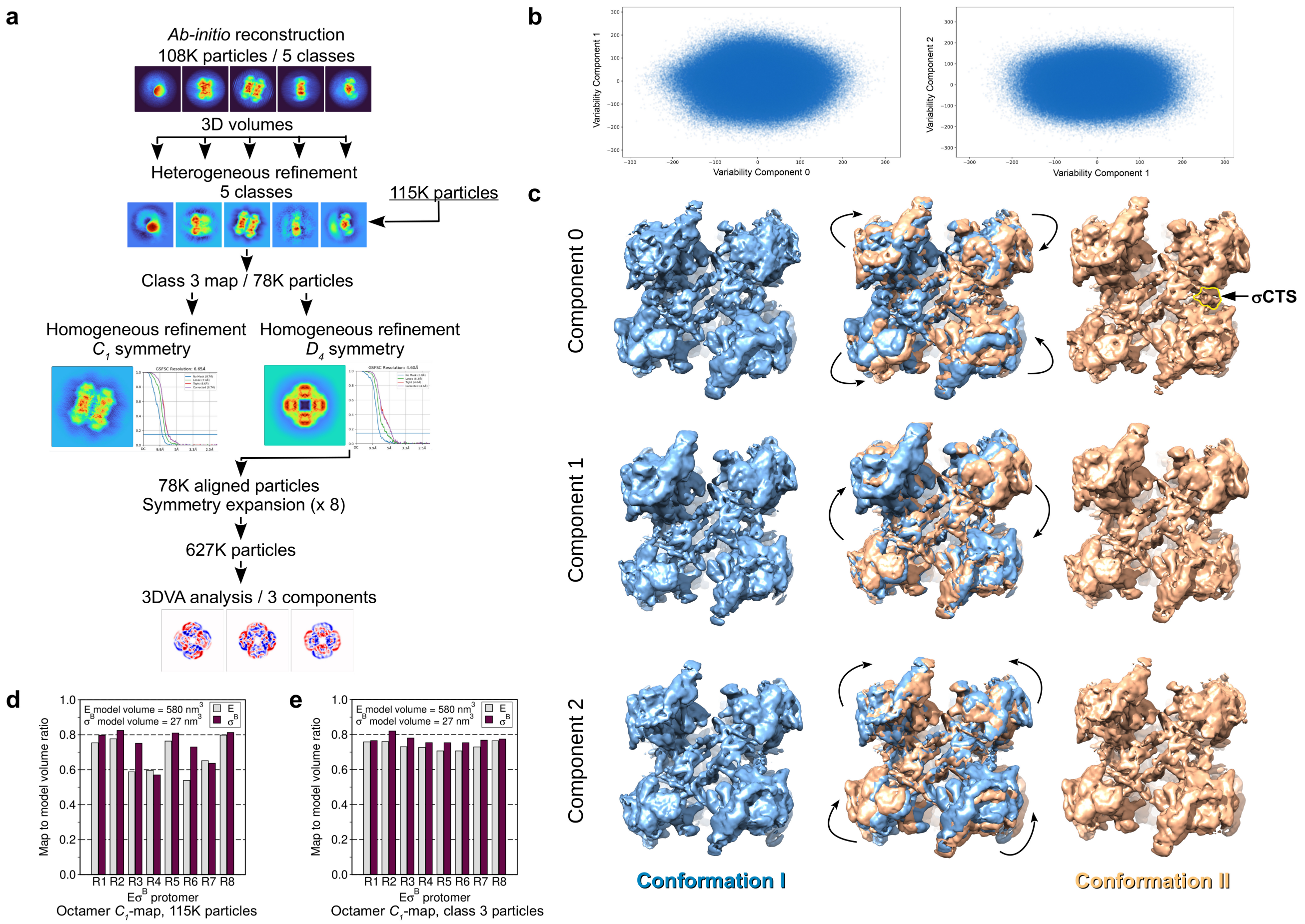

Supplementary Figure 5. Conformational heterogeneity of the Eσ^B octamer. a Workflow of the 3D variability analysis (3DVA) performed in cryoSPARC. First, a set of 105K particles was used in reference-free *ab-initio* heterogeneous reconstruction to produce five initial models. Next, a dataset of 115K particles and five initial models were used as input for 3D heterogeneous refinement aimed to remove junk particles and to select a clean stack of particles of the RNAP octamer. Afterwards, the class 3 particles and map were used in homogeneous refinement with imposed D_4 symmetry. Aligned particles from the homogeneous refinement job were symmetry expanded and used in 3DVA with 3 components (panels **b, c**). **b** 2D-scatter plots of particles coordinates distribution over 3 components. **c** 3D density maps generated by 3DVA along each variability component. Density of the σ^B C-terminal segment (σCTS) is indicated by arrow and by yellow contour line. **d, e** Bar graphs show relative volumes of the $\sigma^B{}_2$ domain and core RNAP densities in R1 - R8 protomers of the octamer C_1-map. Values are the ratios of the map volume to the volume of the molecular model. Cryo-EM maps were refined using a full set of 115K particles (**d**) and cleaned set of 78K particles (**e**) derived from the heterogeneous refinement job shown in panel (**a**). Volumes were calculated in UCSF Chimera after the splitting the Cryo-EM maps by Segger[2]

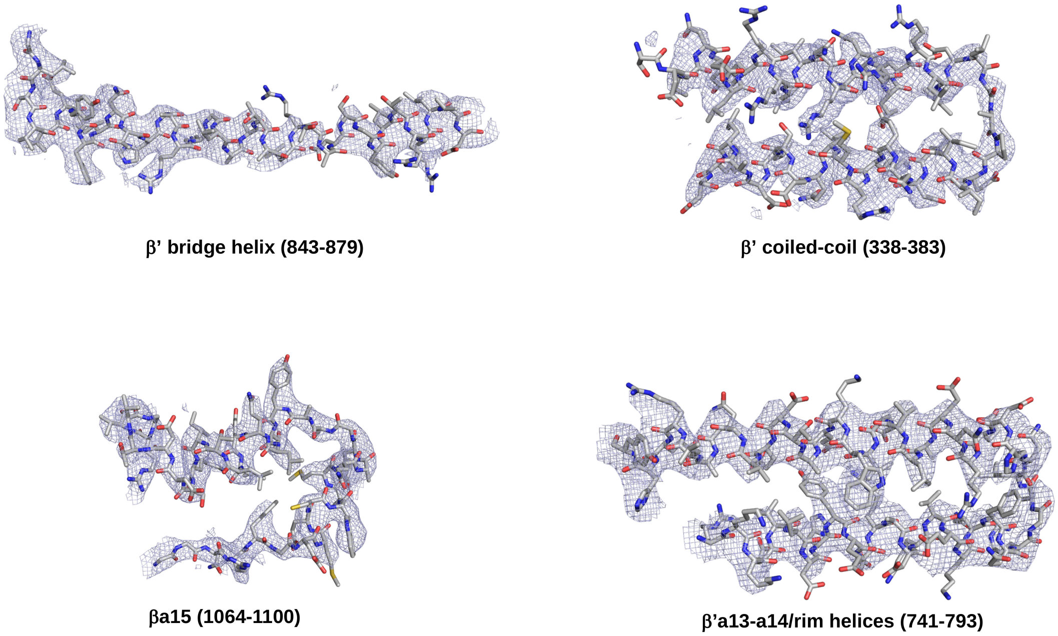

Supplementary Figure 6. ***M. tuberculosis*** **Eσ^B R1 protomer.** Sample density maps and molecular models of the *M. tuberculosis* Eσ^B structural elements.

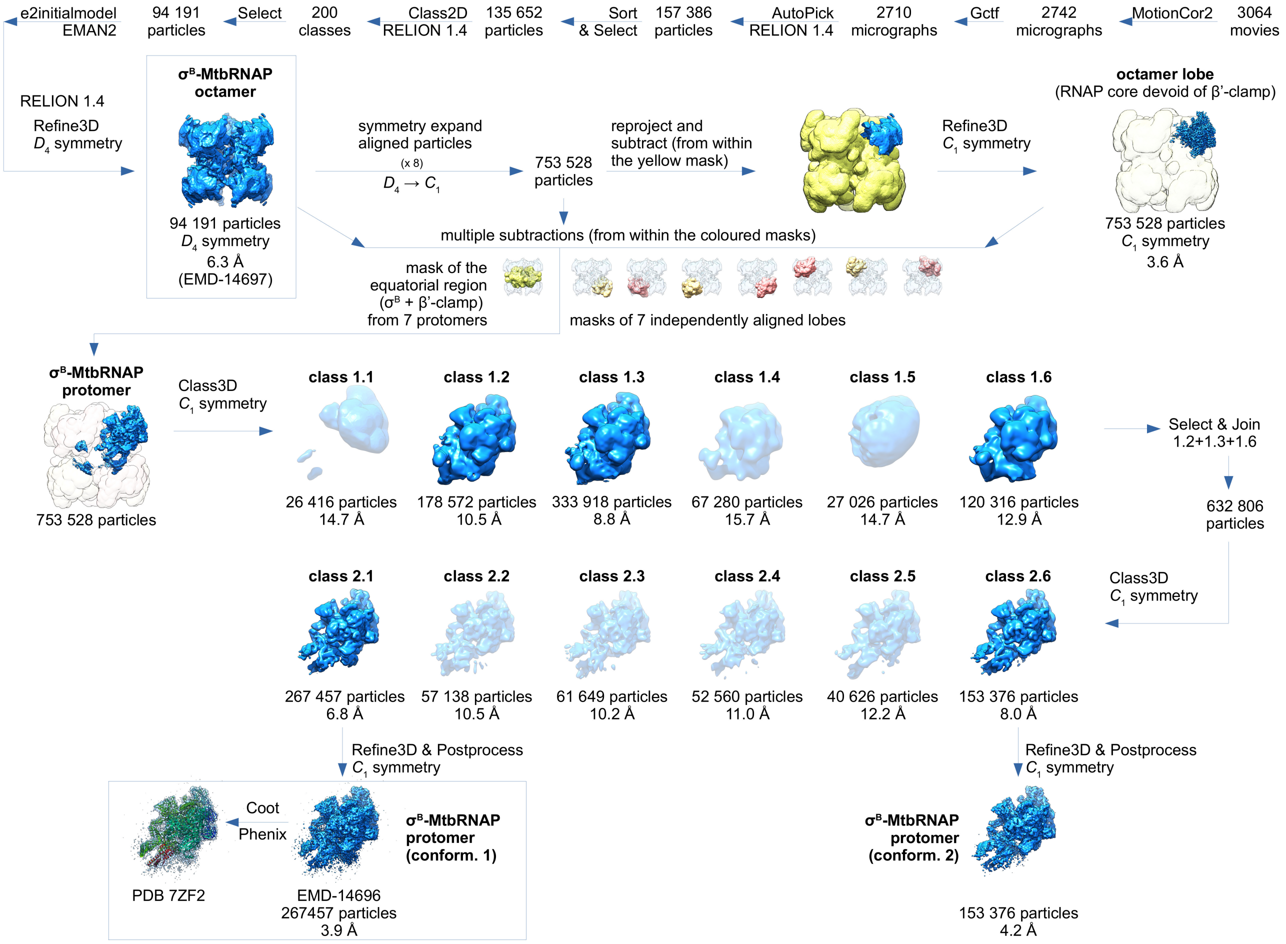

Supplementary Figure 7. Alternative cryo-EM data processing pipeline for *M. tuberculosis* Eσ^B using RELION

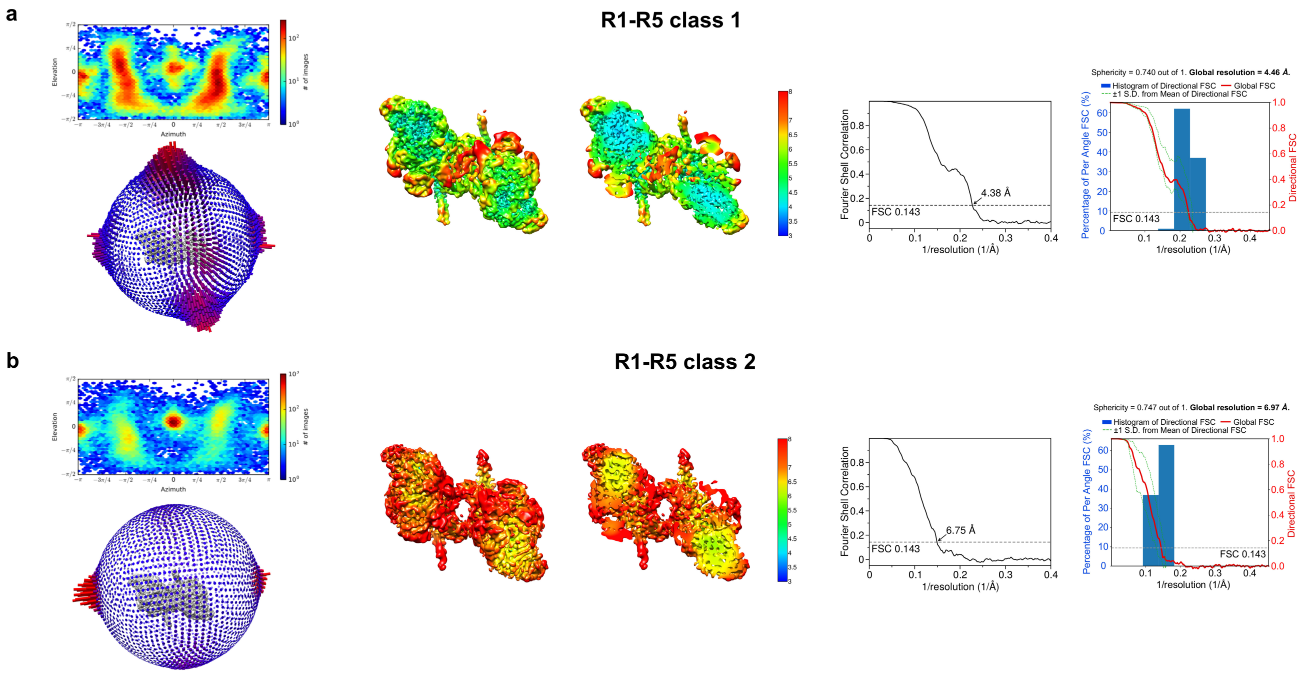

Supplementary Figure 8. Two conformations of the Eσ^B dimer. a Eσ^B R1-R5 dimer conformation I (3D class 1). **b** Eσ^B R1-R5 dimer conformation II (3D class 2). From left to right: angular distributions for particles projections calculated in cryoSPARC and presented as a heat map and as a sphere. Cryo-EM density maps and sliced maps (on the right) colored according to the local resolution. Gold-standard FSC calculated for the map in cryoSPARC. Dotted line shows the 0.143 FSC cutoff. Histogram (blue) and directional FSC Plot (red line) calculated for the cryo-EM map using on-line application at https://3dfsc.salk.edu/[1]. Gold-standard FSC calculated for the model vs. full-map using the MTRIAGE module of Phenix (right). The dotted line shows the 0.5 FSC cutoff.

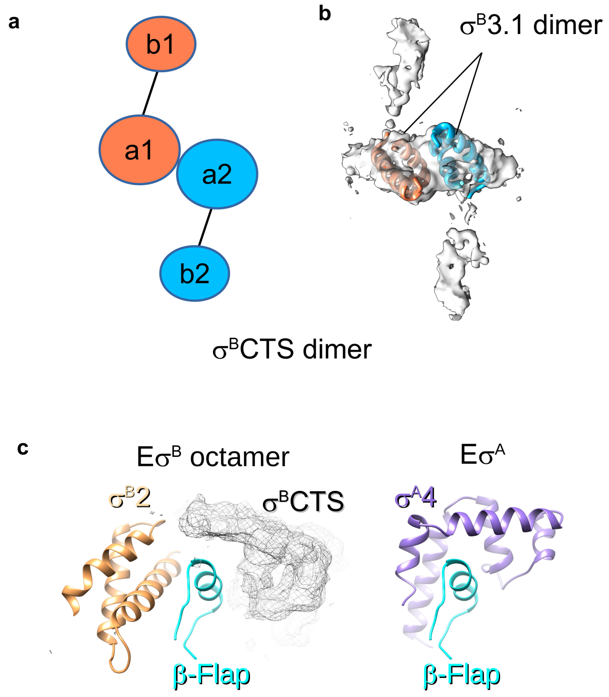

Supplementary Figure 9. The C-terminal segment of σ^B. **a** Schematic representation of the two stacked σ^B C-terminal segments (σ^B CTS) in the R1-R5 (Eσ^B)$_2$ dimer. Each σ^{B}CTS is depicted as two connected ellipsoids. The σ^{B}CTS assigned to the R1 RNAP protomer is colored in coral (a1-b1) and the σ^{B}CTS assigned to the R5 RNAP protomer (a2-b2) is in deep sky blue. **b** Cryo-EM density of the two stacked σ^{B}CTS in the R1-R5 (Eσ^B)$_2$ dimer with the fitted model of the σ^B subregion 3.1 dimer (residues 162-212, shown as ribbons). The σ^B monomers are colored as in panel B. **c** Environment of the β flap in the Eσ^B octamer (left) and in Eσ^A (right). The unresolved density of the σ^B C-terminal segment (σ^{B}CTS) is shown as a mesh. The σ^B domain σ2 and the σ^A domain σ4 are shown as ribbons.

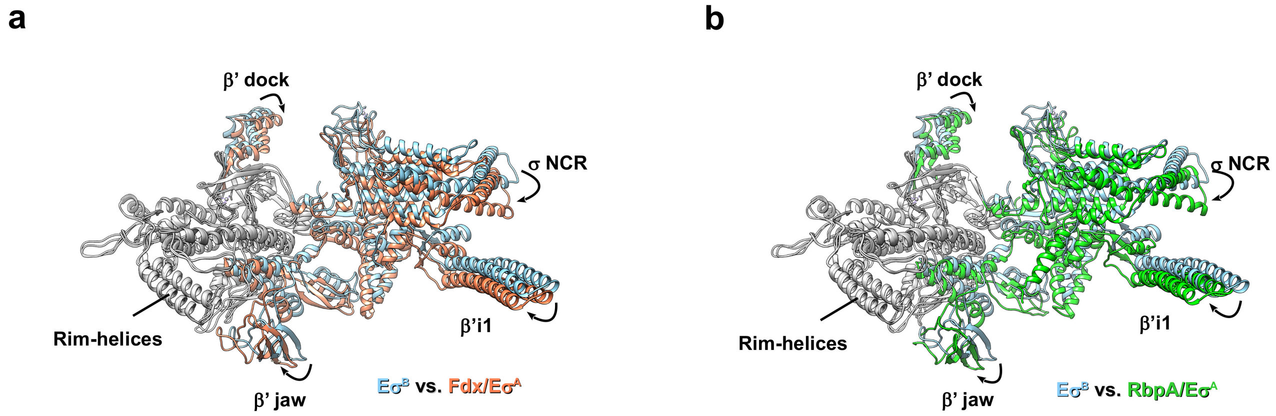

Supplementary Figure 10. Conformational changes in Eσ^B. **a** Superposition of the β' subunit domains: β'i1 (a.a. 141-230), β'jaw (a.a. 1037-1116), β' dock (β'a11, aa 440-495)[3], and σ^BNCR in Eσ^B with those of Eσ^A in complex with the antibiotic Fdx (PDB ID 6FBV). **b** Superposition of the β' subunit domains: β'i1 (a.a. 141-230), β'jaw (a.a. 1037-1116), and σ^BNCR in Eσ^B with those in RbpA/Eσ^A (PDB ID 6C05).

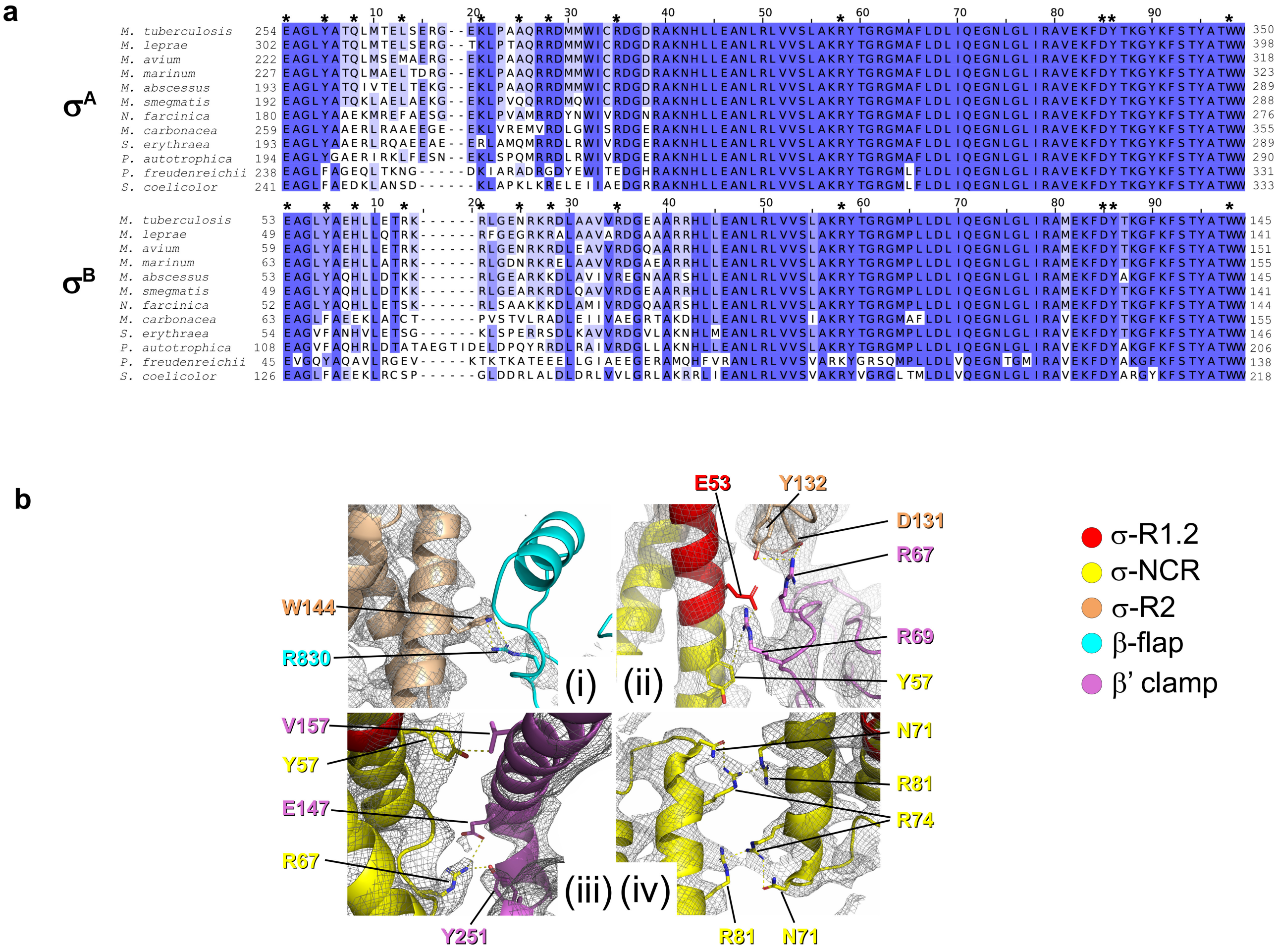

Supplementary Figure 11. Structure of the octamer-forming interfaces. a Alignment of the representative sequences of the σ^B and σ^A subunits of *Actinobacteria* implicated in interface formation. Asterisks show residues implicated in inter-subunit interactions. Residues are shaded blue according to the blosum62 information score. **b** Views on the inter-subunit interfaces: (i) β flap - σ^B, (ii) β'ZBD - σ^B (iii) β'i1 - σ^B, (iv) σ^BNCR - σ^BNCR. The cryo-EM density is shown as a gray mesh with the superimposed ribbons color coded as indicated on the right. Interacting residues are shown as sticks and labeled.

Supplementary References

1. Tan, Y. Z. *et al.* Addressing preferred specimen orientation in single-particle cryo-EM through tilting. *Nat. Methods* **14**, 793–796 (2017).
2. Pintilie, G. D., Zhang, J., Goddard, T. D., Chiu, W. & Gossard, D. C. Quantitative analysis of cryo-EM density map segmentation by watershed and scale-space filtering, and fitting of structures by alignment to regions. *J. Struct. Biol.* **170**, 427–438 (2010).
3. Lane, W. J. & Darst, S. A. Molecular Evolution of Multisubunit RNA Polymerases: Structural Analysis. *J. Mol. Biol.* **395**, 686–704 (2010).
