# Supplementary material for: Structural basis of the mycobacterial stress-response RNA polymerase auto-inhibition via oligomerization
Source: Nat Commun. 2023 Jan 30;14:484. doi: 10.1038/s41467-023-36113-y (PMC9886945; doi:10.1038/s41467-023-36113-y)
Supplement: Supplementary file 3 — Description of Additional Supplementary Files [file 41467_2023_36113_MOESM3_ESM.pdf]

Description of Additional Supplementary Files

File Name: Supplementary Movie 1
Description: Oscillation between 3D density maps generated by 3DVA in cryoSPARC. Variability component 0 is shown. Movie was generated in USCF Chimera.
