# Supplementary material for: Structural basis of the mycobacterial stress-response RNA polymerase auto-inhibition via oligomerization
Source: Nat Commun. 2023 Jan 30;14:484. doi: 10.1038/s41467-023-36113-y (PMC9886945; doi:10.1038/s41467-023-36113-y)
Supplement: Supplementary file 5 — Reporting Summary [file 41467_2023_36113_MOESM5_ESM.pdf]

nature portfolio

Corresponding author(s): Konstantin Brodolin and Patrick Bron

Last updated by author(s): Dec 22, 2022

Reporting Summary

Nature Portfolio wishes to improve the reproducibility of the work that we publish. This form provides structure for consistency and transparency in reporting. For further information on Nature Portfolio policies, see our Editorial Policies and the Editorial Policy Checklist.

Statistics

For all statistical analyses, confirm that the following items are present in the figure legend, table legend, main text, or Methods section.

n/a	Confirmed	
☐	☒	The exact sample size (*n*) for each experimental group/condition, given as a discrete number and unit of measurement
☐	☒	A statement on whether measurements were taken from distinct samples or whether the same sample was measured repeatedly
☒	☐	The statistical test(s) used AND whether they are one- or two-sided<br>*Only common tests should be described solely by name; describe more complex techniques in the Methods section.*
☒	☐	A description of all covariates tested
☒	☐	A description of any assumptions or corrections, such as tests of normality and adjustment for multiple comparisons
☐	☒	A full description of the statistical parameters including central tendency (e.g. means) or other basic estimates (e.g. regression coefficient) AND variation (e.g. standard deviation) or associated estimates of uncertainty (e.g. confidence intervals)
☒	☐	For null hypothesis testing, the test statistic (e.g. *F*, *t*, *r*) with confidence intervals, effect sizes, degrees of freedom and *P* value noted<br>*Give P values as exact values whenever suitable.*
☒	☐	For Bayesian analysis, information on the choice of priors and Markov chain Monte Carlo settings
☒	☐	For hierarchical and complex designs, identification of the appropriate level for tests and full reporting of outcomes
☒	☐	Estimates of effect sizes (e.g. Cohen's *d*, Pearson's *r*), indicating how they were calculated

Our web collection on statistics for biologists contains articles on many of the points above.

Software and code

Policy information about availability of computer code

Data collection	EPU 2 Software (ThermoFisher)
Data analysis	Motioncor2, Gctf v1.06, EMAN2, cryoSPARC 2, RELION 1.4, Modeller, UCSF Chimera 1.13, PyMOL 1.8, Coot 0.8.9, Phenix 1.19, ImageQuant TL 7.0, Grace 5.1.25, Weblogo 2.82

For manuscripts utilizing custom algorithms or software that are central to the research but not yet described in published literature, software must be made available to editors and reviewers. We strongly encourage code deposition in a community repository (e.g. GitHub). See the Nature Portfolio guidelines for submitting code & software for further information.

Data

Policy information about availability of data

All manuscripts must include a data availability statement. This statement should provide the following information, where applicable:

- Accession codes, unique identifiers, or web links for publicly available datasets
- A description of any restrictions on data availability
- For clinical datasets or third party data, please ensure that the statement adheres to our policy

The publicly available datasets with PDB IDs: 6FBV [https://doi.org/10.2210/pdb6fbv/pdb], 6EYD [https://doi.org/10.2210/pdb6eyd/pdb], 6C04 [https://doi.org/10.2210/pdb6c04/pdb], 6C05 [https://doi.org/10.2210/pdb6c05/pdb], 6KON [https://doi.org/10.2210/pdb6kon/pdb], 3WOD [https://doi.org/10.2210/pdb3wod/pdb], 6EDT [https://doi.org/10.2210/pdb6edt/pdb] were used in this study for data analysis and figure preparation. Cryo-EM density maps reported in

March 2021

this paper have been deposited in the Electron Microscopy Data Bank with accession codes EMD-13579 [https://www.ebi.ac.uk/emdb/EMD-13579] (Mtb EsigB protomer), EMD-14696 [https://www.ebi.ac.uk/emdb/EMD-14696] (Mtb EsigB protomer), EMD-13817 [https://www.ebi.ac.uk/emdb/EMD-13817] (Mtb EsigB octamer D4-map), EMD-14697 [https://www.ebi.ac.uk/emdb/EMD-14697] (Mtb EsigB octamer D4-map), EMD-13829 [https://www.ebi.ac.uk/emdb/EMD-13829] (Mtb EsB dimer), EMD-14378 [https://www.ebi.ac.uk/emdb/EMD-14378] (Mtb EsB dimer class 1), EMD-14974 [https://www.ebi.ac.uk/emdb/EMD-14974] (Mtb EsigB dimer class 2), EMD-14560 [https://www.ebi.ac.uk/emdb/EMD-14560] (Mtb RNAP core). Model coordinates have been deposited in the Protein Data Bank with accession codes 7PP4 [https://doi.org/10.2210/pdb7PP4/pdb] (Mtb EsigB protomer), 7ZF2 [https://doi.org/10.2210/pdb7ZF2 /pdb] (Mtb EsigB protomer), 7Q4U [https://doi.org/10.2210/pdb7Q4U/pdb] (Mtb EsigB octamer), 7Q59 [https://doi.org/10.2210/pdb7Q59/pdb] (Mtb EsigB dimer), 7Z8Q [https://doi.org/10.2210/pdb7Z8Q/pdb] (Mtb RNAP core). Source data are provided with this paper.

Human research participants

Policy information about studies involving human research participants and Sex and Gender in Research.

Reporting on sex and gender	N/A
Population characteristics	N/A
Recruitment	N/A
Ethics oversight	N/A

Note that full information on the approval of the study protocol must also be provided in the manuscript.

Field-specific reporting

Please select the one below that is the best fit for your research. If you are not sure, read the appropriate sections before making your selection.

☒ Life sciences ☐ Behavioural & social sciences ☐ Ecological, evolutionary & environmental sciences

For a reference copy of the document with all sections, see nature.com/documents/nr-reporting-summary-flat.pdf

Life sciences study design

All studies must disclose on these points even when the disclosure is negative.

Sample size	Sample size (number of particles) in Cryo-EM was defined by the available time of access to microscope and by the aim to achieve highest resolution. Sample size in biochemical assays was chosen on the basis of the decades of laboratory practice to guarantee the reliability of the results. Number of samples in kinetic experiments was determined on the basis of the time interval required for reaction completion.
Data exclusions	Particle images were excluded during the data processing in cryoSPARc and RELION. Exclusion was done on the basis of standard criteria applied by the data processing software
Replication	The biochemical experiments were performed in duplicates and triplicates at controlled conditions which allow reproducibility of the results. All attempts at replication were successful. Structure reconstructions performed using two different programs (RELION and cryoSPARC) gave consistent results
Randomization	In cryo-EM 3D refinement, all particles were randomly split into two groups used to calculate gold standard Fourier Shell Correlation. Randomization is not relevant to biochemical experiments reported in this study which do not include clinical trials, experiments on live organisms or live cells. All experiments were performed in duplicates and triplicates at controlled conditions with no need in allocation to a particular experimental group.
Blinding	Blinding is not relevant to our study. Data collection and quantification was performed in automatic mode using dedicated equipment which exclude subjective bias. Biochemical experiments were independently reproduced by two team members using at least two different preparations of proteins.

Reporting for specific materials, systems and methods

We require information from authors about some types of materials, experimental systems and methods used in many studies. Here, indicate whether each material, system or method listed is relevant to your study. If you are not sure if a list item applies to your research, read the appropriate section before selecting a response.

Materials & experimental systems

n/a	Involved in the study
☒	☐ Antibodies
☒	☐ Eukaryotic cell lines
☒	☐ Palaeontology and archaeology
☒	☐ Animals and other organisms
☒	☐ Clinical data
☒	☐ Dual use research of concern

Methods

n/a	Involved in the study
☒	☐ ChIP-seq
☒	☐ Flow cytometry
☒	☐ MRI-based neuroimaging
